# The Puzzle of Genetic Stability and Chromosomal Copy Number Alterations for the Therapy of Ewing Sarcoma

**DOI:** 10.3390/cancers17223719

**Published:** 2025-11-20

**Authors:** Günther H. S. Richter, Andreas Ranft, Maximilian Kerkhoff, Marvin Jens, Ina E. Kirchberg, Uta Dirksen

**Affiliations:** 1 Department of Pediatrics, Division of Oncology and Hematology, Charite-Universitätsmedizin Berlin, 13353 Berlin, Germany; marvin.jens@charite.de; 2 German Cancer Consortium (DKTK), Partner Site Berlin, 13353 Berlin, Germany; 3 Pediatrics III, University Hospital Essen, West German Cancer Center, 45147 Essen, Germany; andreas.ranft@uk-essen.de (A.R.); maximilian.kerkhoff@uk-essen.de (M.K.); inaelisa.kirchberg@uk-essen.de (I.E.K.); 4 German Cancer Consortium (DKTK), National Center for Tumor Diseases (NCT) Site Essen, 45147 Essen, Germany

**Keywords:** Ewing sarcoma, EWSR1 haploinsufficiency, loss of heterozygosity, replication stress, biomarker, replication stress directed therapy

## Abstract

Studies of the genomic stability of Ewing sarcoma (EwS) have produced contradictory findings. While they are generally characterized by low mutation rates of individual genes, several cases exhibit genomic alterations that manifest as chromosomal gains and losses. Taken together, these alterations represent independent biomarkers for EwS, such as loss of heterozygosity (LOH) or an altered genome. Patients with primary EwS tumors with fewer than three copy number alterations (CNAs) have a better prognosis than those with more CNAs. The functional mechanisms underlying this chromosomal instability are not yet clear. However, there are indications that this may be directly caused by the *EWSR1::ETS* translocations that are characteristic of EwS. The transcriptional behavior of the chimeric transcription factor EWSR1-FLI1 leads to the formation of R-loop DNA–RNA hybrids that form when RNA binds back to DNA during transcription and increased replication stress, which may result in structural chromosomal changes. Additionally, the formation of EWSR1 fusion genes in EwS results in the loss of one or both wild-type EWSR1 alleles in sarcoma cells. As chromosome segregation has been observed under loss of wild-type EWSR1, EWSR1 loss has been proposed as a potential source of LOH. So, it is highly probable that a chromosomal translocation and the subsequent formation of the EWSR1-ETS fusion protein cause the genomic alterations in EwS. This indicates that targeted therapy should be directed against the CNA and LOH biology caused by the fusion protein.

## Simple Summary

Ewing sarcoma (EwS) is a highly malignant tumor of bone and soft tissue that predominantly affects children and young adults, with a high propensity for early metastasis to the lungs and bones. Large-scale sequencing and SNP array studies have concluded that, in addition to the typical *EWSR1::ETS* translocation, the genome of EwS is remarkably simple at the single-nucleotide level, but recurrent structural chromosome alterations are frequent. The current findings indicate that translocation-mediated EWSR1 haplo-insufficiency results in replication stress and potential loss of heterozygosity and emphasize that copy number dysregulation acts as a critical secondary driver of EwS biology, shaping tumor evolution and influencing clinical outcome. The preclinical results directed at replication stress and diminished repair capacity of EwS are promising and should significantly improve the therapy of EwS.

## 1. Introduction

Ewing sarcoma is a highly aggressive malignancy of bone and soft tissue affecting children, adolescents, and young adults [1,2]. Thanks to multimodal treatment—including polychemotherapy combined with surgery and/or radiotherapy—approximately 70–80% of patients with localized EwS can be cured [3]. However, around 25% of patients present with metastatic disease already at the time of diagnosis. These patients face a significantly poorer prognosis, with survival rates of 20–25% [4]. These data underscore the urgent need for optimized, risk-adapted, and targeted therapeutic approaches, particularly to improve outcomes for high-risk patients.

The molecular hallmark of EwS is a balanced chromosomal translocation that fuses the N-terminus of EWSR1 (Ewing sarcoma RNA-binding protein 1) on chromosome (Chr) 22q12 with an ETS family transcription factor, most commonly FLI1 on Chr 11q24, creating the potent chimeric oncoprotein EWSR1-FLI1. This rearrangement both generates a novel gain of function driver and causes haploinsufficiency of EWSR1, since one wild-type (WT) allele is disrupted by the translocation. The question addressed here is how loss or functional attenuation of the remaining EWSR1 allele intersects with, or even promotes, the broader spectrum of copy number alterations (CNAs) and loss of heterozygosity (LOH) events that accumulate during EwS evolution. This review summarizes experimental, cytogenetic, and sequencing evidence to support the following arguments: (1) loss of function (LOF) of the EWSR1 gene may promote chromosomal instability (CIN); (2) CIN is the mechanistic basis for the LOH or CNAs in entire chromosomes or chromosomal segments; (3) gain of function of chimeric EWSR1-ETS proteins promote replication stress (RS) and probably foster CIN; and (4) recurrent secondary lesions (e.g., STAG2, CDKN2A and TP53) emerge within the CIN-permissive context. Collectively, these factors influence clinical behavior and serve as pivotal starting points for the development of new targeted therapies.

## 2. Normal Function of EWSR1

EWSR1 is a multifunctional protein involved in RNA processing, regulation of gene expression, and maintenance of genome stability [5]. It is not classified as a transcription factor [6]. EWSR1 is located on Chr 22q12.2 and encodes a ubiquitously expressed RNA-binding protein. The EWSR1 protein belongs to the FET (FUS, EWSR1, TAF15) family of RNA-binding proteins [7]. It contains an N-terminal transcriptional activation domain (TAD), an overlapping prion-like domain, and a domain rich in glutamine, serine, and tyrosine (QSY), as well as a C-terminal RNA-binding domain with RNA recognition motifs (RRMs) and arginine–glycine–glycine (RGG) repeats. It interacts with RNA polymerase II and various transcription factors to modulate gene expression [8]. EWSR1 participates in pre-mRNA splicing, mRNA export, and stability. It is further involved in genome integrity through its interactions with DNA Repair-Associated Protein BRCA1 and BRCA1-Associated Ring Domain 1 (BARD1) [9]. EWSR1 can form biomolecular condensates via its prion-like domain, which is thought to regulate its transcriptional and RNA-processing roles [10,11]. EWSR1 associates with α-tubulin and stabilizes spindle microtubules; depletion prolongs prometaphase and increases the probability of unaligned chromosomes [12].

Interestingly, EWSR1 also interacts with Aurora B kinase (AURKB) and helps localize it to the inner centromeres and spindle midzone. AURKB is the catalytic kinase of the chromosomal passenger complex. It phosphorylates histone H3 (Ser10) and coordinates kinetochore–microtubule attachment, spindle checkpoint signaling, and cytokinesis. Inhibition or acute depletion causes lagging chromosomes, multipolar spindles, aneuploidy, and apoptotic/mitotic death [13,14]. EWSR1 further associates with R-loops [15]. R-loops consist of an RNA-DNA hybrid and displaced single-stranded DNA (ssDNA) and are ubiquitous in organisms ranging from bacteria to mammals [16]. R-loops have been described at DNA replication initiation sites in bacteria. They appear to regulate various cellular processes, such as gene expression, immunoglobulin class switching (Ig), and DNA repair [16]. Kabeche et al. reported that ATR (ataxia–telangiectasia and Rad3-related protein) is associated with R-loops at the centromere during mitosis [17].

A recent study found that EWSR1 binds centrosomal RNA and centromere protein A (CENP-A) via R-loops in the centrosome region and is necessary for the maintenance of centromeres in interphase [18]. Most eukaryotes have large arrays of repetitive DNA in their centromeres. However, it is believed that the inheritance of the centromere is primarily related to epigenetic modifications. CENP-A, the centromeric variant of histone H3, is considered an important epigenetic marker [19,20]. Following DNA replication, centromeric nucleosomes, including the existing CENP-A, are evenly distributed to the sister chromatids. Newly synthesized CENP-A is then deposited at the centromere during the early G1 phase of the cell cycle in humans [21]. This regulation is crucial for the proper function and inheritance of the centromere [19]. The authors observed that CENP-A physically interacts with centromeric RNA in a manner dependent on EWSR1. The SYGQ2 region within the prion-like domain of EWSR1 appears to be necessary for binding to CENP-A. EWSR1 binds to R-loops of this RNA, and CENP-A deposition at the centromere depends on these R-loops [18]. Collectively, these activities position EWSR1 as a guardian of mitosis and chromosomal stability.

## 3. Role of EWSR1 Gene Fusions in Ewing Sarcoma and Other Tumors

EwS is defined by specific balanced chromosomal *EWSR1::ETS* translocations, leading to chimeric proteins that function as aberrant transcription factors and determine the complex and highly malignant phenotype [2,22]. The most well-characterized fusion is EWSR1-FLI1 (t(11;22)(q24;q12)) in ~85% of EwS cases. Other partners include ERG, FEV, ETV1, and others, most from the ETS transcription factor family.

Fusions involving EWSR1 occur in other tumors, including desmoplastic small round cell tumor (EWSR1-WT1), clear cell sarcoma (EWSR1-ATF1), myxoid liposarcoma, extra-skeletal myxoid chondrosarcoma, and rare CNS tumors [23]. These fusions retain the EWSR1 N-terminal TAD that functions as a strong transcriptional activator [24] fused to the DNA-binding domain of the partner transcription factor.

Notably, in addition to simple reciprocal translocations, typical *EWSR1::ETS* gene fusions can also arise from chromoplexy. This phenomenon is characterized by large-scale chromosomal chain rearrangements. Chromoplexy is a form of abnormal DNA repair, an alternative mechanism to simple reciprocal translocation, which is observed not only in EwS but also in other translocation-associated sarcomas and may potentially serve as a prognostic marker [25,26]. Chromoplexy affects a few rearranged regions, in the range of ten chains scattered across many chromosomes, rather than being limited to one or two chromosomes. Analysis of the breakpoint sequences showed that all partner fragments involved in these serial rearrangements originate from double-strand breaks (DSBs) and exhibit a deletion at the fusion sites of the chained rearrangements, with EWSR1 at the center of these rearrangements. The biological consequences of this coordinated multigene disruption remain poorly defined. The initial results on 124 patients indicated chromoplexy-positive EwS are enriched for TP53 mutations, had more relapses versus non-chromoplexy cases, and were associated with a more aggressive form of the disease [25]. The most recent information obtained by Dermavan and colleagues using targeted DNA and RNA sequencing analyzing 173 patients confirmed chromoplexy in 31% of cases, primarily validating the driving fusion event and the presence of secondary genetic alterations for risk stratification [26], but they found similar rates of TP53, STAG2, and CDKN2A genetic alterations and arm-level CNAs between cohorts with or without chromoplexy, although a higher incidence of chromoplexy in metastatic patients at diagnosis was observed [26]. Furthermore, the study similarly noticed a higher incidence of chromoplectic chains in EWSR1::ERG tumors (42%) compared to EWSR1::FLI1 (24%) [26]. Future investigations have to figure out whether these chromoplectic events, after all, are significantly associated with a poorer patient prognosis.

The fusion proteins can act both as potent aberrant transcription factors [27], driving expression of oncogenic targets (e.g., *NR0B1*, *CCND1*) [2] and as transcriptional repressors, depending on the sequence of DNA binding sites and the presence of additional co-factors [27,28]. EWSR1-FLI1 disrupts normal chromatin structure and induces changes in the 3D genome architecture [27,29], and it can phase separate and form transcriptional condensates with co-factors (e.g., BRD4) and RNA polymerase II [30].

EWSR1-FLI1 acts directly or indirectly on many important cellular processes, such as cell cycle, apoptosis, angiogenesis, metabolism, and cell migration, by binding to these sites [2]. EWSR1-FLI1 binds to DNA either at ETS-like consensus sites with a GGAA core motif or at GGAA microsatellites (GGAA-mSats). EWSR1-FLI1 multimers directly induce open chromatin at GGAA-mSats by recruiting the nucleosome remodeling BRG1/BRM-associated factor complex (BAF) and establishing de novo enhancers that interact with promoters to drive gene expression [27,29]. Fusion multimers physically interact with BAF complexes, which appear to be critical for EWSR1-FLI1 function, as BAF complexes are required for the activation of EWSR1-FLI1 target genes. The variable length of GGAA-mSats in the germline may lead to differential activity of these enhancers and is an important determinant of tumor progression [31].

Conversely, EWSR1-FLI also binds to canonical ETS recognition sites without repeats and represses wild-type ETS factors, which can lead to the suppression of enhancers and downregulation of nearby genes [27]. The chimeric transcription factor can directly repress certain genes, such as *LOX* and *TGFBR2*, through direct interaction and recruitment of the nucleosome remodeling and the deacetylase repressor complex (NuRD), which includes histone deacetylases and the histone demethylase LSD1 [32].

Thus, the initiating role of a chromosomal aberration leading to the formation of the *EWSR1::ETS* fusion driving the disease is well established, as are many of the downstream consequences of fusion expression on transcriptional and epigenetic regulation. This review aims to broaden the scope by highlighting additional effects of fusion formation, which directly or indirectly increase the likelihood of DNA damage accumulation, specifically copy number alterations and loss of heterozygosity.

## 4. EWSR1-FLI1—Driven R-Loop Accumulation

In 2018, Gorthi and colleagues reported that EwS cells display altered regulation of damage-induced transcription and R-loop accumulation, as well as increased RS [15]. Herein, they demonstrated that EWSR1-FLI1 enhances transcriptional output, leading to R-loop formation and RS in EwS cells. Wild-type EWSR1 suppresses the activation of RNA polymerase II, while EWSR1-FLI1 promotes it, even when WT EWSR1 is present. Biochemical data indicate that this occurs via the regulation of cyclin-dependent kinase 9 (CDK9) and association with BRCA1. As early as 2003, it was demonstrated that BRCA1 associates with the active transcription complex and dissociates from it after DNA damage [33]. BRCA1 encodes a tumor suppressor protein that plays a central role in preserving genomic stability. It functions primarily in the repair of DNA DSBs through homologous recombination (HR) by coordinating with BRCA2, the partner and localizer of BRCA2 (PALB2), and recombinase RAD51 [34]. BRCA1 has been shown to physically interact with CDK9, a kinase best known for its role in transcription elongation as part of the P-TEFb complex. Functionally, CDK9 co-localizes with BRCA1 at DNA DSB sites following ionizing radiation, and its depletion impairs BRCA1 and RAD51 focus formation, leading to defective HR repair [35]. Without EWSR1 or in the presence of EWSR1-FLI1, damage-induced global transcription is not properly halted, and BRCA1 fails to translocate to the site of DNA damage [15], thereby impairing homologous recombination. BRCA1 is not only retained at canonical target sites but also in EWSR1-FLI1-driven genes in EwS, further limiting BRCA1 availability. As a result, HR in EwS is impaired due to increased interaction between BRCA1 and the elongating transcription apparatus. In a similar manner, hyperactive or aberrant transcription by EWSR1-FLI1 results in increased R-loops at active replication loci, blocking corresponding replication forks and potentially triggering RS [15,36]. On the other hand, Gorthi and colleagues also observed that EWSR1 plays a role in the transcriptional response to damage by suppressing R-loops and promoting HR [15].

However, the following question remains: How does EwS cope with endogenous RS caused by increased R-loop formation? Recently, Koppenhafer and colleagues discovered that EwS cells rely on ATR and checkpoint kinase 1 (CHEK1) signaling [36]. When activated by DNA RS, the ATR-CHEK1-WEE1 pathway coordinates a multi-faceted response that arrests the cell cycle, suppresses origin activation, stabilizes replication forks, and promotes fork repair and restart [37]. In addition, ATR and CHEK1 also fulfill important and unique functions outside of the S phase and DNA RS response. They regulate chromosome segregation, the S-G2 checkpoint, the G2-M transition, the repair of DNA DSBs, and the response to osmotic and mechanical stress [17,37]. Here, the G2 checkpoint kinase WEE1 phosphorylates and inhibits the cyclin-dependent kinases CDK1/2 to enforce the S/G2-M checkpoints. Blocking WEE1 leads to premature mitosis and cell death when DNA is damaged or under-replicated.

It was further observed that EWSR1-FLI1 prevents the resolution of R-loops induced by DNA topoisomerase-1 poisons by sequestering DHX9 helicase, which can ultimately lead to R-loop accumulation, RS, and genomic instability. Conversely, overexpression of DHX9 or reduced EWSR1-FLI1 levels has been observed to render EwS cells resistant to the active metabolite of irinotecan (SN-38), independent of proliferation and global transcription rates, helping to explain why elevated DHX9 levels predict poorer clinical outcomes [38].

Overall, BRCA1 function and HR are compromised in EwS, shifting DNA repair toward error-prone pathways [15]. RS is a major driver of structural CIN in general, producing focal and arm-level CNAs, chromosome-scale losses, and copy-neutral LOH (CN-LOH) through fork collapse and error-prone repair [39,40,41]. However, direct evidence that R-loop accumulation at certain chromosomal loci in EwS results in CNA so far is lacking.

## 5. Does EWSR1 Loss Directly Promote LOH/CNAs?

A previous study showed that knocking out ewsa (the zebrafish ortholog of human EWSR1) in zebrafish leads to chromosome segregation defects due to mitotic dysfunction [42]. Chromosome exchange occurs during metaphase when sister chromatids align. EWSR1 has been shown to translocate the mitotic regulator AURKB to the midzone. The midzone is essential for maintaining spindle architecture, spindle elongation, and furrow formation [43,44]. Defects in cytokinesis resulting from the inability to relocate AURKB kinase to the midzone are associated with uneven chromosome separation and the induction of aneuploidy [45]. A subsequent zebrafish study generated ewsa null mutants on a p53+/− background that drastically increased the incidence of tumorigenesis [46]. Tumors that occurred in ewsa−/−; tp53+/− fish showed LOH of the remaining WT tp53 allele, which was confirmed by restriction fragment length polymorphism and sequencing analysis. In contrast, tp53+/− fish without ewsa loss rarely lost the second allele [46]. The same study documented widespread mitotic errors in ewsa-deficient embryos and cells, supporting a causal chain from EWSR1 loss to genomic instability.

Using an auxin-inducible degron in near-diploid human DLD-1 cells, acute EWSR1 depletion led to lagging chromosomes, mislocalized AURKB, and elevated aneuploidy rates within a single mitotic cycle. Rescue with WT EWSR1 reversed aneuploidy, whereas an AURKB binding-defective mutant failed to do so, demonstrating here that EWSR1’s interaction with AURKB is mechanistically required to prevent CIN [13].

Cytogenetic and CGH/FISH analyses in EwS [47,48] and related EWSR1 fusion tumors (e.g., desmoplastic small round-cell tumors) have reported cases where the formation of *EWSR1*-fusion genes in EwS results in the loss of one or both WT *EWSR1* alleles in the sarcoma cells^.^ [47,48] or where normal Chr 22 is lost and the derivative Chr 22 is duplicated, effectively eliminating the WT EWSR1 allele (complete LOH at 22q12) [49]. While anecdotal and not yet quantified in large cohorts, these findings suggest a selective advantage for complete WT EWSR1 loss during tumor evolution, consistent with the zebrafish and cell line data. Further biological circumstances that also need to be considered for LOH induction are that loss mitotic function of EWSR1 and the associated potential increase in DNA damage lead to alternative splicing of genes involved in DNA repair and genotoxic stress signaling, including Abelson Murine Leukemia Viral Oncogene Homolog 1 (ABL1), Checkpoint Kinase 2 (CHEK2), and Mitogen-Activated Protein Kinase Kinase Kinase Kinase 2 (MAP4K2) [50]. Therefore, LOH may be triggered by impairment of two EWSR1 functions: mitosis and splicing of DNA repair molecules. Together, they support the hypothesis that EWSR1 haploinsufficiency can drive subsequent CNAs/LOH.

## 6. The Copy Number/LOH Landscape of Ewing Sarcoma

### 6.1. Global Features

Large-scale sequencing and SNP array studies have concluded that the genome of EwS is remarkably simple at the single-nucleotide level, with a mean mutation load of ~0.1–0.2 per Megabase, but recurrent structural chromosome alterations are frequent [22,51,52,53,54]. Already, in 2002, it was shown that over 50% of EwS tumors present with non-random cytogenetic aberrations in addition to the characteristic *EWSR1::ETS* translocation [55]. Patients with EwS presenting fewer than three CNAs have a better prognosis than those with more CNAs [56].

CNAs in EwS often involve arm-level or whole-chromosome gains and losses that can encompass hundreds of genes. These large-scale CNAs likely exert their effects through cumulative dosage imbalances across multiple functionally interacting loci, rather than through isolated single-gene drivers [57]. Among the most recurrent alterations are gain of chromosome 1q frequently coupled with loss of 16q (commonly arising from an unbalanced t(1; 16) translocation) at a frequency of 31–43%, gain of chromosome 8 (~50%), and gain of chromosome 12 (21–26%). Less common, though recurrent, are the focal deletions of 9p21 encompassing *CDKN2A/B* (~12%) [55,58,59,60], as well as whole-chromosome gains of chromosome 20 (~15%) and complex, clustered CNAs in proximity to fusion loci such as *EWSR1* and its partners [25,26]. These typical CNAs, together with their presumed mechanistic basis and biological rationale, are summarized in Table 1.

Mechanistically, several of these CNAs appear to be positively selected during tumor evolution because they buffer replication or mitotic stress induced by the EWSR1::FLI1 fusion. For instance, trisomy 8—observed in approximately half of EwS tumors—increases RAD21 dosage, thereby mitigating *EWSR1::FLI1*-driven replication stress and conferring a proliferative advantage [59]. Similarly, the frequent co-occurrence of 1q gain and 16q loss reflects CIN processes, such as mitotic mis-segregation or breakage–fusion–bridge (BRB) cycles, resulting in a single unbalanced translocation event [55,60]. Trisomy 12, by contrast, represents a recurrent numerical aberration consistent with mitotic mis-segregation and clonal selection across multiple cohorts [58,60].

Longitudinal and phylogenetic analyses indicate that EwS genomes evolve under therapeutic pressure, acquiring additional whole-chromosome or arm-level CNAs during progression and relapse. Primary and recurrent tumors may diverge early and evolve in parallel, suggesting subclonal selection rather than a purely linear evolutionary trajectory [25,62]. Importantly, both the percentage of the genome affected by CNAs and the total number of CNA events correlate inversely with survival, underscoring the role of aneuploidy burden as a key prognostic indicator [56,58,61,63,64,65,66,67]. Together, these findings emphasize that copy number dysregulation acts as a critical secondary driver of EwS biology, shaping tumor evolution, buffering fusion-induced stress, and influencing clinical outcome advantage (see Table 1 for representative CNAs and mechanistic context) [59].

### 6.2. Prognostic Significance

Several of the recurrent CNAs summarized in Table 1 have been evaluated for prognostic relevance. Most of these CNAs were based on retrospectively collected patient material. Careful prospective testing of some of these recurrent CNAs or genetic markers, however, often failed to confirm their prognostic significance. CNAs can be assayed by different methods, including SNP arrays or DNA-FISH, which might theoretically explain some of the discrepancies between studies. However, the disagreement between retrospective and prospective studies seems to hold across different methods used for CNA detection, and in our view, is most likely a consequence of biased cohort composition [68].

In an initial study on 34 EwS patients, Chr 8 gain seemed to occur more frequently at relapse [69], but in two retrospective studies of 134 or 112 EwS patients, wherein gain of Chr 8 was observed in 52% or 47% of cases, respectively, there was no association with survival [52,55]. Subsequent studies suggested a prognostic association of chromosome 8 gain, with 8q24/MYC amplification [70], or increased RAD21 dosage [59], consistent with their role in replication-stress buffering. But, a possible contributing role of a MYC amplification was not mechanistically confirmed [59].

*Cyclin-dependent Kinase Inhibitor 2A* (*CDKN2A/B*) homozygous deletion (Chr 9p21) was observed in ~6–18% of tumors [71], but its prognostic significance for patient survival has been inconsistent in a prospective study of 112 EwS patients [68]. Gain of Chr 12 was associated with worse event-free survival in 26% of patients with localized disease [55], while loss of 16q in 43% of patients significantly correlated with poor overall survival and was linked to metastatic disease at diagnosis. The frequent co-occurrence of Chr 1q gain and Chr 16q loss events in 41% of patients likely arises from a single unbalanced t(1; 16) event reflecting mitotic mis-segregation or breakage–fusion–bridge cycles. This pattern suggests a single unbalanced translocation as the initiating event. Candidate driver genes include genes promoting cell cycle progression, whereas Chr 16q harbors potential tumor suppressors. Importantly, gain of Chr 1q was associated with poor overall survival and event-free survival in all patients, independent of disease stage [55]. Furthermore, Chr 1q amplification, found in 31% of EwS tumor samples, was strongly correlated with relapse and adverse overall outcome, independent of standard risk factors [58], although all the results listed last were based on retrospectively collected patient material.

In a recent, not yet published study, analyzing 300 prospectively collected EwS patient samples Chr 1q gain and Chr 16q loss were analyzed [72]. Remarkably, the large dataset confirmed that Chr 1q gain was associated with worse survival outcomes in patients with localized disease in 24% of cases, but not in metastatic patients. Additionally, CNA profiling was performed on a subset of patient samples using single-nucleotide polymorphism arrays (SNPa) to assess their utility in clinical evaluation and to prospectively investigate the impact of genome-wide CNA profile on prognosis. SNPa analyses in this subset of 140 patient samples revealed that gains were significantly more frequent than losses, identifying recurrent CNA patterns consistent with previous studies [52,54,56,58]. Moreover, overall LOH in 49% of patients, as measured by SNPa arrays, was a strong predictor (hazard ratio (HR) of 2.44 for overall survival (OS)) of clinical outcomes of the studied patients, independent of the initial disease stage.

Both our group and others have demonstrated that the percentage of the genome altered (PGA), which we recently computed from SNPa array data, is associated with poor outcome in EwS [52,54,58]. Kaplan–Meier estimates revealed an unfavorable prognosis for cases with high PGA in localized disease when considering either event-free survival (EFS) or overall survival (OS) as clinical endpoints. In addition, hazard ratio analyses showed that elevated PGA detected in 49% of patients was a predictive factor (HR of 2.24 for EFS), independent of other clinical variables, in the entire series, including patients with initial metastases and was superior to CNA values, such as Chr 1q gain (HR of 1.58 for EFS) or Chr 16q loss (HR of 1.27 for EFS), respectively.

## 7. Recurrent Mutations Affecting Genome Stability

In addition to CNAs, single-nucleotide variants may contribute to genome instability in EwS. Large next-generation sequencing studies revealed recurrent mutations in STAG2 (15–20% of cases) and TP53 (5–10% of cases) [51,52,53,54]. STAG2, a cohesin complex subunit, is involved in maintaining chromosomal stability and facilitating DNA repair. Interestingly, loss of STAG2 protein expression was also observed in non-mutated cases at an additional frequency of ~20%, indicating that additional mechanisms, such as epigenetic silencing, account for STAG2 loss of expression. Patients with localized disease and loss of STAG2 expression, as assessed by immunohistochemistry, show significantly poorer outcomes compared to patients with retained STAG2 expression. This is associated with poorer survival outcomes, independently of other clinical features, making STAG2 loss of expression a promising marker for risk stratification [73]. Although STAG2 loss in EwS does not appear to significantly impact EWSR1-FLI1 transcriptional activity, CTCF/cohesion insulator function, or H3K27 acetylation profiles [74], it may still exacerbate chromatid cohesion defects, further enhancing CIN. The prognostic relevance of TP53 mutation for patient survival remains debated, but the concurrent mutation of STAG2 and TP53 has been associated with particularly poor outcomes, suggesting a highly permissive environment for genomic instability [52].

## 8. Clinical Correlates and Therapeutic Implications

### 8.1. Risk Stratification 

Integrating genomic with clinical variables improves risk resolution in EwS and should inform both intensification and trial allocation. Beyond stage and tumor size, LOH, PGA, Chr 1q gain/16q loss, and dual STAG2/TP53 loss consistently demarcate high-risk patient subsets with inferior outcomes. Notably, PGA and LOH have emerged as independent prognostic indicators for progression, arguing that structural CIN captures biology not reflected by conventional factors. An important next step is to determine whether additional features—such as chromoplexy, which can substitute for simple reciprocal translocation in EwS and other fusion-driven sarcomas—can be integrated into a composite “genomic variation index” that further refines prediction beyond LOH and PGA [25,26]. Such an index could support biomarker-guided recruitment in studies testing RS-based therapies and DNA repair co-targets.

### 8.2. Therapeutic Targeting of RS and Vulnerable DNA Repair

RS is a central driver of structural CIN in EwS, producing focal/arm-level CNAs, chromosome-scale losses, and CN-LOH through replication fork stalling/collapse and error-prone repair [39,41,75]. While no study to date has mapped R-loop hotspots in EwS directly onto CNA/LOH breakpoints, the link is consistent with current models of RS-driven genome remodeling [15,16,17]. Strategies that stabilize AURKB localization or mitigate R-loop accumulation could hypothetically counteract CIN, though direct restoration of WT EWSR1 is unrealistic. Consequently, therapeutic strategies that either stress the replication fork further (checkpoint abrogation, dNTP depletion) or block maladaptive repair (PARP, DNA-PK) offer rational avenues (Table 2).

### 8.3. Checkpoint Axis: ATR, CHEK1, WEE1

ATR is the master kinase responding to stalled replication forks. So, it is not surprising that EwS are sensitive to ATR inhibitors [76] or the combination of ribonucleotide reductase (RNR)—the rate-limiting enzyme in the synthesis of deoxyribonucleotides—and ATR-CHEK1 inhibitors (see Table 2) [36].

Treatment with a single ATR or RNR inhibitor resulted in minor to moderate effects, while their combined administration produced strong synergistic effects. ATR and RNR inhibitors triggered synergistic cell death and acted together to induce mitochondrial depolarization, caspase-3/7 activity, and DNA fragmentation, suggesting an apoptotic form of cell death [85]. Furthermore, ATR inhibition using berzosertib produces potent activity in EwS preclinical models and synergizes with cisplatin when dosing schedules exploit cell-context dependencies [76,77] (Table 2, ATR targeting). Although not effective so far in preclinical models of TP53-mutated EwS, it targets EWSR1-FLI1-induced RS and related ATR dependency and could lead to the use of platinum drugs, which are not currently employed as standard chemotherapeutic agents for EwS, into the therapeutic armamentarium for treatment in the future.

CHEK1 inhibition (e.g., prexasertib) abrogates S-phase checkpoint control and can precipitate replication catastrophe in RS-primed EwS cells, with robust preclinical activity and early clinical signals in translocation-driven sarcomas [78,79] (Table 2, CHEK1 targeting). WEE1 inhibitors (e.g., adavosertib) force premature CDK1/2 activation, exacerbating RS and enhancing DNA damage; pediatric Phase I studies (with irinotecan) confirmed partial responses in EwS [80,81] (Table 2, WEE1 targeting). Together, these data position checkpoint blockade as a backbone to (i) augment platinum sensitivity where cisplatin is not standard and (ii) rationally combine with agents that intensify replication fork collapse.

### 8.4. DNA Repair Axis: PARP and DNA-PK

EWSR1-FLI1-mediated BRCA1 displacement from transcription complexes produces an HR-defective state (“BRCAness”) that sensitizes EwS to PARP inhibitors (PARPi) [15,89]. However, adaptive restoration of HR via tumor protein p53-binding protein 1 (TP53BP1) loss can blunt PARPi efficacy, consistent with modest single-agent activity in relapsed EwS. Combinations that pair PARPi with TOP1 poisons or ATR inhibitors seek to convert transient replication fork stalling into lethal collapse (Table 2, PARP and ATR targeting). Early pediatric/mixed-histology experiences (e.g., olaparib + ceralasertib; AcSé-ESMART, NCT02813135) demonstrate tolerability but limited efficacy, underscoring the need for biomarker-guided enrollment and schedule optimization. In parallel, blocking NHEJ with DNA-PK inhibitors (e.g., peposertib, AZD7648) can prevent DSB repair after replication fork collapse and synergize with PARPi/TOP1i; though EwS-specific clinical activity remains to be established [83,84] (Table 2, DNA-PK targeting).

### 8.5. RS Co-Targets and Source Control

Because RS in EwS arises partly from EWSR1-FLI1-driven hyper-transcription and R-loop accumulation, targeting the source of RS complements checkpoint/repair strategies. Dual ATR and HSP90 inhibition overwhelm RS tolerance independent of TP53 status in EwS models [86] (Table 2, HSP90 co-targeting). Inhibitors of transcriptional CDKs (CDK7/12/13) reduce hyper-transcription and R-loops, thereby enhancing DNA damage and PARPi sensitivity; activity has been demonstrated in EwS models and PDXs [87,88] (Table 2, transcription CDK axis). Additionally, RNR inhibition with triapine depletes dNTP pools, exacerbates RS, and synergizes with CHEK1/WEE1 blockade in EwS systems [36,81,85] (Table 2, RNR targeting). Collectively, these “RS source” and “RS buffering” attacks are complementary and may be most effective when matched to biomarkers of replication fork stress and repair capacity.

### 8.6. Resistance Considerations and Rational Combinations

Persistent BRCA1 mislocalization helps explain initial PARPi sensitivity, whereas TP53BP1 depletion or alternative end-joining can restore HR and confer cross-resistance to chemotherapy and PARPi in EwS [15]. These observations favor combinations that (i) prevent HR restoration (ATR/CHEK1), (ii) block compensatory repair (DNA-PK), or (iii) reduce R-loop burden (transcription-CDK inhibitors). Early pediatric experiences with PARPi+ATRi (AcSé-ESMART) show feasibility but modest activity, reinforcing the need for prospective biomarkers and adaptive schedules (Table 2, PARP/ATR targeting).

Furthermore, it was also demonstrated that EWSR1-FLI1 binds ETS motifs in the *AURKB* promoter and transcriptionally up-regulates AURKA/B in EwS, suggesting a possible compensatory mechanism for the observed chromosomal mislocalization of AURKB (see above), identifying an additional vulnerability [90,91] and providing a rationale for the use of multi-AURKA/B inhibitors (e.g., tozasertib, danusertib). EwS show nanomolar sensitivity, synergy with topoisomerase II inhibitors, and in vivo activity, suggesting combined AURKA/B blockade as a credible adjunct, although selective AURKB inhibitors (e.g., barasertib) have not yet advanced in EwS-specific trials [12,50,90,92].

### 8.7. Cohesin Deficiency and ATR/PARP Dependency

EwS tumors with inactivating STAG2 mutations may experience heightened RS and altered enhancer–promoter architecture, plausibly increasing sensitivity to ATR or PARP pathway inhibition [73,74,93,94]. Whether STAG2 loss exacerbates R-loop accumulation or creates additional synthetic lethalities with DSB-repair genes in EwS warrants dedicated investigation. Such dependencies could furnish patient selection criteria for trials testing ATRi/WEE1i/PARPi combinations.

## 9. Conclusions

Several independent lines indicate a model in which EWSR1 haploinsufficiency and EWSR1-FLI1-induced hyper-transcription promote RS, thereby favoring the limited but recurrent repertoire of CNAs/LOH as observed in EwS. Zebrafish and cell-based systems provide direct evidence that loss of EWSR1 promotes LOH and aneuploidy via AURKB -dependent mitotic defects, while human tumor profiles confirm selection for conditions that allow CIN. In parallel, EwS tumors exhibit a reproducible but limited repertoire of CNAs/LOH—primarily 1q gain, PGA, STAG2, and TP53 alterations—that correlates with poor clinical outcome. The convergence of these data supports a model in which EWSR1 haploinsufficiency is an upstream driver of chromosomal instability that triggers prognostically relevant CNAs/LOH. To achieve clinical benefit, integrative, allele-resolved genomics, functional assays of RS/repair capacity, and schedule-conscious therapy combinations prospectively guided by biomarkers are required. Such efforts should refine risk stratification and significantly improve therapy in EwS.

## Figures and Tables

**Table 1:** Typical CNAs in EwS and their proposed mechanistic basis *.

CNA (Typical Call)	Key Locus/Genes	Likely Mechanism(s)	Why This Mechanism Possibly Fits/Assumed Correlative Evidence	References
+1q with −16q (often together)	1q (multiple), 16q (multiple)	Unbalanced translocation der(16)t(1; 16) results in arm-level gain/loss	Classic cytogenetic/CGH work shows 1q gain frequently co-occurs with 16q loss via der(16)t(1; 16) at ~31–43%; reproduced across cohorts and summarized in consensus data	[55,60]
+8 (whole chromosome) ± 8q24 focal amp	RAD21, MYC(8q24)	Mitotic mis-segregation/selection(aneuploidy) + replication-stress buffering; high-level MYC amplification via double minutes/BFB in a subset	~50% EwS harbor +8. Functional work shows +8 is selected because extra RAD21 dampens EWSR1-FLI1—induced replication stress; occasional MYC high-level amplifications are consistent with BFB/episomal amplification	[59]
+12 (whole chromosome)	multiple (MDM2/CDK4 region not typically amplified in EwS)	Mitotic mis-segregation/selection (aneuploidy)	Trisomy 12 is a recurrent numerical change at ~21–26% across CGH/array studies, consistent with whole-chromosome mis-segregation and clonal selection	[55,60]
9p21 focal deletion (homozygous in subset)	CDKN2A/B	Focal DSB/repair-mediated deletion under selection (replication stress → DSBs → resection/micro-HR/NHEJ)	Recurrent focal loss of CDKN2A at ~12% across EwS cohorts; pattern is focal rather than arm-level, consistent with local break/repair and strong selective pressure	[52,58]
+20 (whole chromosome)	multiple	Mitotic mis-segregation/selection	Recurrent in ~15% of patients, whole-chromosome gain across CGH/array cohorts	[58,61]
Complex, clustered focal CNAs near fusion loci (subset)	regions around EWSR1 and partners	Chromoplexy-linked rearrangement bursts (with passenger CNAs)	~31–42% of EwS harbor chromoplexy forming around the fusion; the same bursts generate additional SVs and local CNAs early in tumor evolution	[25,26]

* The mechanisms and connections presented here are a proposed model based on indirect and correlative evidence and are not yet fully proven or linear pathways. BFB: breakage–fusion–bridge as a cyclic process of chromosome breakage and rejoining that generates CNAs.

**Table 2:** Therapeutic strategies exploiting replication-stress biology in Ewing sarcoma.

Mechanistic Class/Target	Representative Agents	Mechanism of Action	Rationale in EwS (EWSR1 Loss/EWSR1-FLI1 RS)	Evidence in EwS (Status)	Key Refs.
Checkpoint Axis					
ATR	VE821, Berzosertib (M6620)	Inhibits ATR, the master kinase sensing stalled forks	R-loops and fork stalling sensitize to ATR block	Potent single-agent activity in EwS cell lines and xenografts; schedule-optimized cisplatin+ATRi synergy. Clinical development is ongoing in solid tumors	[76,77]
CHEK1/CHEK2	Prexasertib (ACR-368)	Abrogates the S-phase checkpoint; results in replication catastrophe	RS + high CDK activity creates checkpoint addiction	Robust preclinical data; pediatric Phase I (combo with irinotecan), a confirmed PR in an EwS pt; tumor-agnostic trials (ongoing)	[78,79]
WEE1	Adavosertib (AZD1775), ZN-c3	Forces CDK1/2 activation; results in premature mitosis/DNA damage	EwS cells rely on WEE1 to cope with RS; combining with RNR or TOP1i is rational	Preclinical synergy with RNRi; pediatric Phase I (with irinotecan) with PR in EwS	[80,81]
DNA-Repair Axis					
PARP1/2	Talazoparib, Olaparib, Niraparib	Inhibits PARP1/2; traps PARP on DNA	BRCA1 displacement by EWSR1-FLI1 causes HR defect; synergy with TOP1i or ATRi	Limited single-agent efficacy; combination trials (e.g., with irinotecan; with ATRi)	[15,82] (AcSé-ESMART)
DNA-PK (NHEJ)	Peposertib (M3814), AZD7648	Blocks DNA-PKc-mediated DSB repair (NHEJ)	Fork collapse yields DSBs; NHEJ inhibition increases lethality	Preclinical rationale; early-phase data in solid tumors; clinical activity in EwS not yet established	[83,84]
RS Co-targets/Source Control					
RNR	Triapine	Depletes dNTPs, exacerbating RS	EwS depends on adequate dNTP pools; synergy with CHEK1/WEE1 inhibition	Preclinical EwS data show activity of triapine ± WEE1i; RRM2 depletion links to ATR/CHEK1 block	[36,81,85]
HSP90	Luminespib (AUY922 ± ATRi)	Destabilizes client proteins; ATRi + HSP90i intensifies DDR	Dual inhibition overwhelms RS tolerance independent of TP53	Preclinical synergy in EwS models	[86]
Transcriptional CDKs (CDK7/12/13)	THZ1, THZ531 (prototypes)	Suppresses hyper-transcription and R-loop formation	EWSR1-FLI1-driven RS reduced; enhances DNA damage/PARPi efficacy	Preclinical sensitivity; PARPi combination active in PDX models	[87,88]

Legend: ATRi, ATR inhibitor; DDR, DNA damage response; HR, homologous recombination; NHEJ, non-homologous end joining; PARPi, PARP inhibitor; RNRi, ribonucleotide-reductase inhibitor; RS, replication stress; TOP1i, topoisomerase-I inhibitor. “Evidence” summarizes EwS-specific preclinical/clinical status drawn from cited sources.
